# Interfacing analytical instruments to electronic data processing systems: a summary of problems

**DOI:** 10.1155/S1463924681000412

**Published:** 1981

**Authors:** Heiko Pangritz

**Affiliations:** Hahn-Meitner-Institut für Kernforschung Postfach 39 01 28 Berlin 39 D-I000 Germany


### Interfacing analytical instruments to electronic data processing systems: a summary of problems

**Heiko Pangritz**

*Hahn-Meitner-Institut für Kernforschung, Postfach 39 01 28, D-1000 Berlin 39, W. Germany.*

Laboratory data processing in clinical chemistry and haematology consists of:

1. Registration of requests, specimen preparation and sample distribution;
2. Data acquisition, calculation of results, and evaluation of series of data, for instance for quality control purposes; and
3. Data presentation.

This paper will cover only some aspects of item (2) of this list.

The program system of a central laboratory computer includes a number of tasks, which become active only at that moment when measuring results from the analytical instruments or other input devices arrive at the laboratory computer. Among the functions of those program sections are for instance the calculation of final results, the association of the results with the patient data, the compilation and presentation of reports, the evaluation of quality control measurements and others. All these functions can be fulfilled without significant difficulties and without remarkable effort in realtime programming, if all information is made available to the computer in the form of complete and self describing data blocks.

A very simple example of such a data block is given in the recommendations of the German Society for Medical Documentation, Informatics, and Statistics from 1975 concerning the hardware interfaces of analytical instruments [1]. This example is shown in Figure 1.

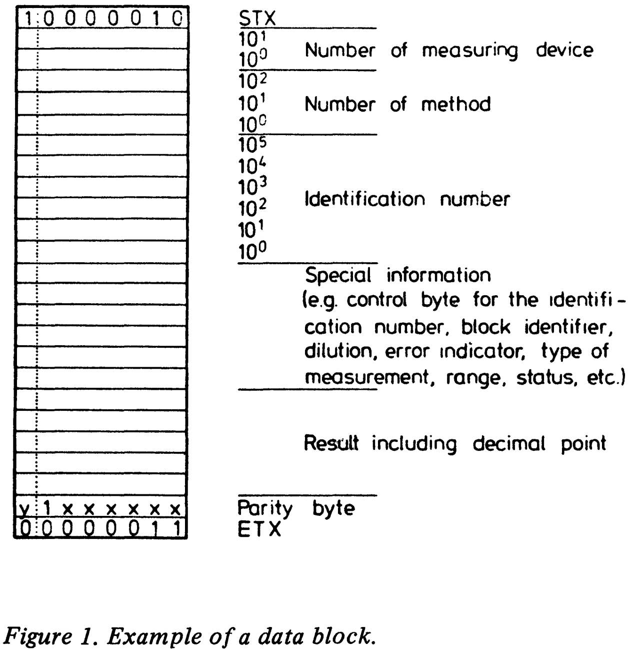

*Figure 1. Example of a data block.*

This data block includes the number of the device, the number of the method or test, the sample identification number, some additional information on the status and operation of the measuring instrument, and finally the actual result of the test.

The task of connecting an analytical instrument with the central data processing system of a laboratory is essentially to collect, in one way or the other, these different items of information forming the data block.

Figure 2 shows a basic structure of functions and devices with regard to the on-line connection of analytical instruments to a computer. This figure does not refer to the implementation of any of the shown functions. The basic structure comprises on one side the laboratory computer with its data management facilities and on the other side the actual analytical instrument, a component for organisation and calculation tasks, a device for reading the sample identification number, and a device for preparing additional data records for safety and backup reasons. Other possible and sometimes desirable components like a data terminal with input keyboard are not shown here in order to simplify the picture.

From the viewpoint of interface problems obviously the ideal case is that when the interface between the measuring device and the computer is actually at point A in Figure 2, the measuring device itself delivers the wanted information block and implements the other functions such as data recording and sample identification. But this case is not realistic if it is necessary to calculate final results taking into account factors and parameters which cannot be known by the measuring device. For instance, the calculation of a creatinine clearance with regard to the creatinine values in serum and urine. The volume and collecting period of the urine, as well as height and weight or body surface of the patient can only be handled by the central computer. Also, only with very few analytical devices it is possible to preset charge dependent factors for the calculation of final results, another task which must be performed by the central computer. It is thus realistic to have the interface between measuring device and central computer at point B in Figure 2, where the calculation of final results is reserved for the computer. Also according to Figure 1 a block of information is transferred via this interface, and in most cases the results in those blocks will be the final test results.

The configuration with interface B (shown in solid lines) in Figure 2 contains, as a part of the analytical instrument or as an additional device, a component able to make a strict correlation between sample identification numbers (read at an arbitrary time before the measurement) and their affiliated test values coming from the analytical instrument. This component must also be capable of collecting the necessary additional information on the device number, the method number and so on, to build up an information block as described above and transfer it to the computer.

For several laboratory systems, preprocessing units were developed for this purpose, which were tailormade for particular analytical instruments and unfortunately in most cases also tailormade for the computer system used. Whether one regards those additional components as part of the laboratory computer or as part of the measuring device is insignificant here, as long as the functions described above are fulfilled.

With many laboratory systems a configuration is implemented which follows the dash-point-lines in Figure 2. Here the organisational tasks in connection with the collection of the items of an information block are transferred into the computer hardware and the program system. In this last mentioned configuration the analytical instrument, the preprocessing unit and the data recorder are one unit. If preprocessing of measuring values means for instance calculation of a kinetic from several measuring points or consideration of dilution factors and amplification ranges, and if data recording means the enumerated output of results on a printer, then most modern analytical devices will fulfill these requirements.

In this configuration, measuring device and reader of the sample identification number are connected to different interfaces of the computer. Since the data set being transferred from the measuring device to the computer is no longer complete in the sense of the example given above, there is in practice a rigid relationship between the computer interface and the device. Thus only the combination of the information being received at the interface C of the computer with the information at interface D is allowed and gives a valid information block. The arbitrary connection of devices with computer interfaces in the case of a hardware breakdown in the coupling elements is no longer possible without a reconfiguration of the program system.

The following section of this paper will deal with the contents of the information blocks in greater detail and with the problems of getting the information.

The generation of a device number as an address of origin of an information block presumes that this number can be preset at the analytical device, which in most cases is not possible. Therefore this device number is often preset in the device orientated interface units or is derived by the computer itself from the number of its own interface, with which the measuring device is connected. This last mentioned possibility is very inflexible, but it is used in most cases.

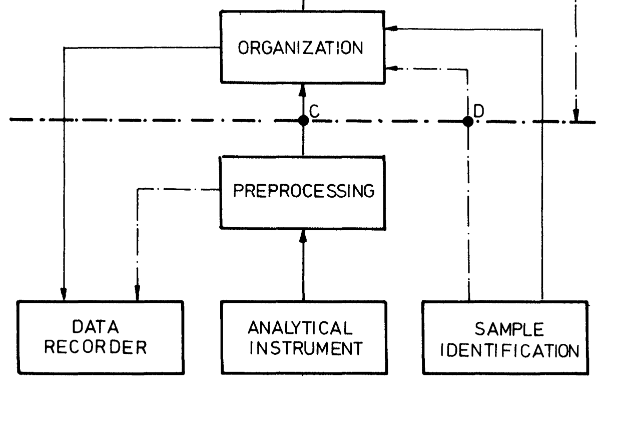

*Figure 2. Basic layout of functions and instrumentation.*

Even more complicated are the problems with the method number. An agreed nomenclature of methods in clinical chemistry and haematology does not exist, nor is there any kind of decimal classification either on a national or international basis. The consequence is that every laboratory invented its own special number system for characterising the different methods. If analytical instruments prefix their test results with a method number, it can thus be taken that it is not the number used in the laboratory system. So, if method numbers according to the laboratory system are to be inserted in the described information blocks, there has to be a 'code transformation' in the device orientated preprocessing units. It is now standard practice to transfer this job to the central computer which, from the number of the interface, the device dependent markings of the test results, or from the sequence of incoming results, derives the method number used in the laboratory.

This paper does not cover details of the problems connected with direct sample identification, but it is known that there is a decreasing number of different identification systems. These systems can be used with more or less effort, but nobody will claim to have made any fundamental progress. In addition there are a great number of users who renounce direct sample identification and rely with sample processing strictly on the order of a working list prepared by the computer. Nevertheless, if some kind of direct sample identification is used, there ought to be a device capable of storing a number of identification numbers read in advance, since only with very few methods and instruments is it possible to read the identification number off the sample container at the time of the measurement. Very often the sample is preprocessed within the instrument, which excludes the transfer of the sample container to the measuring position. The task of storing and allocating identification numbers is seldom fulfilled by the analytical device itself, more often by a device orientated preprocessing unit, and unfortunately in most cases by the laboratory computer itself.

The complete information block described in Figure 1 includes additional information concerning the status of the measuring device, device settings, used factors etc. An interesting facility would be a code word designating the operator at the measuring device. Although the central computer would not require all of the information for the evaluation of the measurement, since most of the analytical instruments handle this part of data processing themselves, it would be desirable to have this information for reasons of laboratory organisation, operational supervision and safety.

But the problem with this additional information is that it can only be gained easily in very few cases. At this point and in this context, a fundamental statement should be made, which should be written over the desk of everybody starting a laboratory data processing project:

> "It is strongly recommended that hardware modifications of any kind made to an analytical instrument in order to enable the on-line connection of this instrument to a data processing system be avoided."

If an analytical device in the commercially available form does not present the information necessary at any kind of port, then it is unfit for on-line data processing. Such a device should only be integrated into a data processing system by off-line data input via terminal and keyboard.

It must be admitted that there is sometimes a great temptation not to follow this recommendation. Take as an example a flame photometer which presents test results at a printer interface. The digital display of the instrument presents the result including decimal point, which depends on the position of a range switch. The printer interface gives just four figures in parallel, without a decimal point, without information on the position of an imaginary decimal point, and without information on the setting of the range switch. A short piece of wire would be an ugly but effective solution of this problem, raising the following questions:

– What does the manufacturer say to this manipulation in connection with servicing the instrument?
– What happens in case of an instrument failure and the attempt to use another device of the same kind?

Summing up this problem it should be said again with emphasis: from the viewpoint of data processing, laboratory organisation, operational supervision and safety it is desirable to get additional information on the operation of different measuring devices. Nevertheless such information should be only used if it is readily furnished by the analytical instrument.

With all existing data processing systems in laboratories the greatest development cost has been associated with incompatible data processing interfaces of individual analytical instruments. It is known that there have been intensive and to some extent fruitful efforts to reach a standardisation in this area, and the new German Standard DIN 66 258 [2] is a good example. But it must be pointed out that an agreement between manufacturers on a small spectrum of standardised interfaces should just be regarded as a first step, and that it would be desirable to develop some basic rules for information contents and information formats and so on. This would further simplify the connection of measuring devices with data processing systems.

Experience shows that some manufacturers are willing to adopt existing standards for the configuration of data processing interfaces, but there is a lack of information on the specific problems of organising a complex laboratory data processing. Consider a case where it is not possible to process one sample out of a series of samples due to mechanical or analytical reasons. If the analyser then just skips this sample without supplying a special signal or a dummy information block at the data processing interface, then the most severe mistakes can be generated, if wrong sample identification numbers are combined with the following test results.

The standardised interfaces recommended for use with analytical instruments in clinical laboratories are all intended for information transfers in both directions. The extent to which this facility is used is not an interface problem but one which depends solely on the design of the instrument, or of the measuring device. From the data processing viewpoint there are several situations in which information from the computer fed back to the measuring device should influence the operation of the instruments. If for instance a first evaluation of quality control measurements shows that an instrument is out of control, it can be stopped and advice concerning the necessary operational steps can be given. In general, modern data processing systems in laboratories are capable of maintaining a dialogue with the operator to present evaluated test results in different contexts and to accept provisional or final confirmations of results and so on. It should be considered whether this possibility, which would give greater power of decision to the operator, should not be used to make work more attractive despite the large amount of flow production.

*This is the first in a series of three papers. The remaining papers will appear in future issues of the Journal.*

REFERENCES

[1] Porth, A. J., Killian, K. and Pangritz, H. "Hardware-Schnittstellen fur den On-line-Anschluβ von Geraten im klinisch-chmischen Labor an Datenverarbeitungsanlagen." Deutsche Gesellschaft fur Medizeinische Dokumentation, Informatik und Statistik e.V. (GMDS), deutsch/englisch, 31 p, Dezember 1975.

[2] Entwurf DIN 66 258 Teil 1: "Schnittstellen und Steuerungsverfahren für die Datenubermittlung." Normenausschuβ Informationsverarbeitung (NI) im DIN Deutsches Institut für Normung eV, Beuth Verlag, Berlin, Juli 1979.

### Awaiting publication in the Journal of Automatic Chemistry

An automated titration system

Present sample identification systems

An assessment of the validity of error flags generated by the Technicon SMAC system and the Perkin-Elmer KA 150 enzyme analyser

Continuous monitoring using thiocyanate ion-selective electrodes

An evaluation of the Kokak glucose/BUN analyser including experience with the NCCLS protocol PSEP-2, 3 and 4

Evaluation of the Technicon Star system for the estimation of serum T4 and T3 uptake

A microcomputer controlled thermostat

Microcomputer control and data system for automated multiple flow injection analysis

A readily available source of serum for use as a precision quality control material for ionised calcium measurement by ion selective electrode

Control programming without language: automation of vitamin B6 analysis

